# The Diagnosis of Gonococcus Infection

**Published:** 1899-09-09

**Authors:** J. O. Symes

**Affiliations:** Bacteriologist, Bristol Royal Infirmary


### HOSPITAL CLINICS AND MEDICAL PROGRESS.

### THE DIAGNOSIS OF GONOCOCCUS INFECTION.

By J. O. Symes, M.D.Lond., Bacteriologist, Bristol Royal Infirmary.

The detection of the gonococcus has become a matter of increasing importance in view of recent researches demonstrating how wide a rôle this organism plays in the causation of disease. That it is the cause of a specific urethritis is now well established, and common secondary infections from the urethra are prostatitis, cystitis, bubonic abscesses, septic arthritis, conjunctivitis, &c. In woman acute or chronic suppurative and inflammatory processes of the uterus, fallopian tubes, and peritoneum may be the result of gonorrhœal infection.

Bennecke and Clement Lucas[1] have more recently demonstrated that joint disease secondary to purulent ophthalmia is not uncommon in infants, and from the fluid of the affected joints the gonococcus may be isolated. Of twenty-three cases recorded by Lucas eighteen were secondary to attacks of ophthalmia neonatorum, whilst the remaining five were cases of ophthalmia caused by secondary inoculation from urethral discharge. In joint affections, as indeed in all secondary gonorrhœal lesions, the gonococcus may be found alone or in conjunction with other cocci.

Thayer, Sydney, and Lazear[2] have shown that gonorrhœa may be the starting point of a septicæmia, due either to the specific organism or to a mixed infection. The same writers have published a series of thirty-two cases illustrating the fact that the gonococcus is not infrequently the cause of serious organic heart lesions. Alone or in conjunction with other cocci it may be the determining cause of a transient or permanent endocarditis, or of the still more grave condition—ulcerative endocarditis. Similarly the pericardium or myocardium may be attacked, and post-mortem examination may reveal small necroses and patches of purulent infiltration in the heart muscle. The methods employed to detect the gonococcus may be classed under two heads —(1) The examination of fluids in cover-glass preparations, by means of special stains; (2) the cultivation of the organism on selected nutrient media.

In any urethral discharge there will be found a large variety of cocci (Foulerton[3] describes eighteen distinct species), and some of these so closely resemble the gonococcus that they have been named pseudococci, and some authorities maintain that it is not possible to distinguish by microscopical examination the true from the false. It has, indeed, been claimed that the gonococcus is to be found in the normal urethra, but this has been refuted by Heiman.[4]

To the experienced observer the recognition of the gonococcus is rendered possible by the combined tinctorial and cultural methods.

In preparing films the procedure is as follows: A drop of the fluid to be examined is flattened out between two cover-glasses, which are then drawn apart, dried, and fixed in the flame. One of the cover-slips is then stained with warm methylene-blue solution for twenty minutes, and the second stained by Gram's method. In specimens prepared with methylene blue the gonococci are seen as diplococci, kidney-shaped, with the flattened borders facing one another, arranged in groups of eight, sixteen, thirty-two, &c., lying within the cells, and stained a deep blue-black. As the gonococcus is decolorised by Gram the second specimen should show no such groups. For a differential stain the following Pick-Jacobsohn's solution may be recommended: Saturated alcoholic solution of methylene blue, ℥ viii.; Ziell Neelsen's carbol fuchsin, ℥ xv.; aqua distillata, 20 c.cs. The film remains in the stain for five seconds, and is then washed, dried, and mounted. In specimens so prepared the protoplasm appears red, mucus threads violet, micro-organisms blue, and the gonococcus black or blue-black.

In acute cases gonococci are to be found lying outside the cells,[5] but it is on their symmetrical arrangement within the pus cell that we chiefly rely for purposes of diagnosis. The material to be examined should in all cases be taken as early in the course of the disease as possible. In ophthalmia it may be collected in a platinum loop or on a sterile cotton swab. In urethritis the meatus must be first cleansed with an antiseptic lotion, the pus then squeezed out from the orifice, the first drop rejected, and the second received on a cover glass. In women the specimen is taken from the urethra, or with the help of a speculum from the cervix, using either a swab or loop.

When examining the blood in cases of ulcerative endocarditis, or the fluid in cases of joint affection, it is not possible, as a rule, on account of the small number of organisms present, to detect the gonococcus in film preparations, although several should be prepared. In such cases about 2 c.cs. of blood or fluid should be drawn off from a vein or the affected joint by means of a sterile syringe, and distributed over the surface of plates of nutrient agar or serum-agar, which are then incubated at 36 deg. C., and the resulting colonies examined. The gonococcus does not grow upon simple nutrient agar, and there is much diversity of opinion as to the best medium for its cultivation. Except for the difficulty and expense of securing a sufficient supply the best material would appear to be the coagulated serum of the rabbit.[6] On this the gonococcus grows far more quickly than does any other coccus. In twelve hours its colonies are visible to the naked eye, round, transparent, elevated in the centre, and viscid. It also grows well on human blood serum, or on a mixture of one part serum and two parts ascitic or pleuritic fluid, or of one part ascitic fluid and two parts of peptonised gelatin. Laitinen[7] recommends a mixture of two-thirds blood serum and one-third ascitic fluid, with 9 c.cs. of caustic potash to each 1,000 c.cs. Steimschneider's medium consists of a watery extract of spleen with serum agar.

The blood-agar plates described by Foulerton[8] have, however, the great advantage of being quickly and easily prepared, and are very widely adopted for the cultivation of the gonococcus.

In the centre of an ordinary agar plate there is placed a drop of blood; to this blood is added a loopful of the material to be examined, and the mixture is spread over the surface of the plate by means of a sterile camel's-hair brush or by a loop. Incubated for 48 hours at body temperature, the colonies of gonococci appear as round, semi-transparent discs with undulated margins. The life of such colonies does not usually extend beyond a week.

In conclusion we may summarise what has already been stated as follows: In the early stage of the disease, having regard to the characters, position, and staining reaction of the micrococci, it is possible from a microscopical examination alone to make a diagnosis. Later in the disease, when other organisms are present, it is difficult to form an opinion from microscopical examination alone, and in such cases it is necessary to resort to cultural methods.

[1] Brit. Med. Jour., Jan. 28, 1899. [2] Jour. of Exp. Med., Jan. 1899. [3] Trans. Brit. Inst. Prevent. Med., Vol. I., p. 39. [4] New York Med. Record, May 1, 1898. [5] Drobny. Archiv. für Derm. et Syph., Oct., 1898. [6] Christmas, Annals de L'Inst. Pasteur, 1897, p. 609. [7] Centralb. für Bact., No. 20, 1898. [8] Trans. Brit. Inst. Prevent. Med., Vol. I., 1897.

